# Herbal composition of *Cinnamomum cassia, Pinus densiflora, Curcuma longa* and *Glycyrrhiza glabra* prevents atherosclerosis by upregulating p27 (Kip1) expression

**DOI:** 10.1186/s12906-016-1224-8

**Published:** 2016-07-28

**Authors:** Jung-Jin Lee, Ji-Hye Lee, Won-Kyung Cho, Joo-Hui Han, Jin Yeul Ma

**Affiliations:** 1 Korean Medicine (KM) Application Center, Korea Institute of Oriental Medicine, Daegu, 701-300 Republic of Korea; 2 Department of Pharmacology, Chungnam National University College of Pharmacy, Daejeon, 305-764 Republic of Korea

**Keywords:** Anti-atherosclerotic activity, Vascular smooth muscle cell, Cell cycle, Cyclin-dependent kinase inhibitor, Kiom-18

## Abstract

**Background:**

Kiom-18 is a novel composition of *Cinnamomum cassia, Pinus densiflora, Curcuma longa* and *Glycyrrhiza glabra. Curcuma longa* and *Glycyrrhiza glabra*, which are traditional medicines in Asia, have been reported to demonstrate preventive effects against atherosclerosis; however, they have not yet been developed into functional atherosclerosis treatments. We therefore studied the anti-atherosclerotic effects and possible molecular mechanisms of Kiom-18 using vascular smooth muscle cells (VSMCs).

**Methods:**

To assess the anti-proliferative effect of Kiom-18 in vitro, we performed thymidine incorporation, cell cycle progression, immunoblotting and immunofluorescence assays in VSMCs stimulated by platelet derived-growth factor (PDGF)-BB. In addition, we used LDLr knockout mice to identify the effects of Kiom-18 as a preliminary result in an atherosclerosis animal model.

**Results:**

Kiom-18 inhibited platelet-derived growth factor (PDGF)-BB-stimulated-VSMC proliferation and DNA synthesis. Additionally, Kiom-18 arrested the cell cycle transition of G_0_/G_1_ stimulated by PDGF-BB and its cell cycle-related proteins. Correspondingly, the level of p27^kip1^ expression was upregulated in the presence of the Kiom-18 extract. Moreover, in an atherosclerosis animal model of LDLr knockout mice, Kiom-18 extract showed a preventive effect for the formation of atherosclerotic plaque and suppressed body weight, fat weight, food treatment efficiency, neutrophil count, and triglyceride level.

**Conclusions:**

These results indicate that Kiom-18 exerts anti-atherosclerotic effects by inhibiting VSMC proliferation via G_0_/G_1_ arrest, which upregulates p27^Kip1^ expression.

## Background

The proliferation of vascular smooth muscle cells (VSMCs) is one of the most important pathogenic mechanisms in the formation of atherosclerotic plaque [1, 2]. This abnormal proliferation contributes to cardiovascular disease, including atherosclerosis, balloon angioplasty-induced restenosis, and hypertension [2–5]. VSMC proliferation is regulated by the cell cycle, which is a complex and stepwise process, via the regulation of cyclin-dependent kinase (CDK)/cyclin complexes and CDK inhibitors (CKIs) [6, 7]. Specific phases in cell cycle progression are regulated by the formation of a complex with the catalytic subunit of CDKs [8, 9]. Upon stimulation, the G_1_ phase of quiescent VSMCs leads to the S phase via the regulation of CDK2/cyclin E1 and CDK4/cyclin D1 complexes in addition to CDK inhibitors such as p21, p53, p27 and p16. This progression results in the hyper-phosphorylation of retinoblastoma protein (Rb) and the dissociation of transcription factor E2F, which initiates the transcription of genes required for DNA synthesis [10, 11]. In previous studies, upregulating p21 and p27 expression has been shown to arrest cells at the G_0_/G_1_ phase to prevent the development of atherosclerosis [12–15].

The topic of this study, Kiom-18, is a novel composition of four herbal components: Chinese cinnamon (*Cinnamomum cassia*)*,* Japanese red pine (*Pinus densiflora*)*,* turmeric (*Curcuma longa*) and licorice (*Glycyrrhiza glabra*)*.* These four herbal components of Kiom-18 have been previously reported to exhibit beneficial in vitro and in vivo effects as traditional medicines. *C. cassia*, a traditional Chinese herbal medicine, has been used in herbal formulas, such as Ge-Gen-Tang (Kakkon-to) and Sini Tang, to manage various diseases, including colds, congestion, fever, heart failure and myocardial infarction [16–21]. *P. densiflora*, a plant that is common throughout Asia, has traditionally been used as a tea, food ingredient, or folk medicine [22, 23]. *C. longa* has been widely used as a traditional medicine in Southeast Asia, and curcumin is its major active component [24]. In addition, *C. longa,* Japanese alder (*Alnus japonica*) and Massa Medicata Fermentata (medicated leaven) make up gambigyeongsinhwan (4), which has been used to treat obesity in local Korean clinics; the mechanism of its anti-obesity effect has recently been established [25]. Traditionally, *G. glabra*, a medicinal herb cultivated in several regions of the world, has been used in the treatment of kidney stones, hepatitis C, and cough and stomach ailments [26, 27]. In a recent report, *C. cassia* was demonstrated to have anti-cancer effects, via the bioactive compound cinnamaldehyde isolated from the stem bark; anti-diabetic effects, due to the inhibition of fibronectin, monocyte chemoattractant protein-1 and interleukin-6 by its active components, anti-osteoporosis effects, as found in Jasin-hwan-gagambang (BHH10), a formula that includes *C. cassia*, and anti-inflammatory effects, through the blockage of NF-kB by cinnamaldehyde [28–32]. *P. densiflora* exerts anti-oxidant activity in a variety of applications, anti-cancer activity in human oral squamous cell carcinoma using an oil formulation of *P. densiflora* leaf and hepatoprotective activity by regulating lipid accumulation and oxidative stress in the liver [33–35]. Recently, *C. longa* and *G. glabra* have been reported to have beneficial effects. *C. longa* may be useful as a natural supplement for the treatment of metabolic syndrome: it has anti-diabetic effects by inhibiting angiotensin converting enzyme, and an ethanolic extract exerts a neuroprotective effect in the hippocampus [36, 37]. Moreover, *G. glabra* has hepatoprotective effects in combination with glycyrrhizin and silymarin, which are metabolites of *G. glabra* and milk thistle (*Silybum marianum*), respectively, and anti-tubercular effects for its extract that includes active compounds [38, 39]. Among these individual components of Kiom-18, *C. longa* and *G. glabra* have been reported in many studies to prevent atherosclerosis by the suppression of low-density lipoprotein (LDL) oxidation; however, to date, they have not been developed as supplemental foods for the treatment of atherosclerosis [40–45]. Therefore, we studied the anti-atherosclerotic effects of a novel composition including *C. longa* and *G. glabra* for its potential use in supplementary foods. We also examined the effects on VSMC proliferation and the molecular mechanism of its anti-proliferative action.

## Methods

### Materials and reagents

The bark from *Cinnamomum cassia* (Nees & T.Nees) J.Presl*,* needles from *Pinus densiflora* Siebold et Zuccarini*,* root from *Curcuma longa* Linne, and root from *Glycyrrhiza glabra* Linne were purchased from an herb market in Yeongcheon, Korea. All plant material identities were confirmed by Dr. Ki Hwan Bae of the College of Pharmacy, Chungnam National University (Daejeon, Korea); all voucher specimens have been stored in the herbal bank at the Korea Institute of Oriental Medicine (Daejeon, Korea). Distilled water was filtered through a 0.45-μm membrane filter from ADVANTEC (Tokyo, Japan) before analysis. Phosphate-buffered saline PBS) and fetal bovine serum (FBS) were obtained from HyClone (Logan, UT, USA). Dulbecco’s modified Eagle’s medium (DMEM) was purchased from Lonza (Walkersville, MD, USA). Trypsin/EDTA and penicillin/streptomycin were purchased from Gibco (Grand Island, NY, USA). Anti-phospho-extracellular signal regulated kinase 1/2 (ERK1/2), anti-ERK1/2, anti-phospho-phospholipase C-γ1 (PLCγ1), anti-phospho-p38, anti-p38, anti-phospho-phosphatidylinositol 3-kinase-linked protein kinase B (Akt), anti-Akt, anti-phospho-c-Jun N-terminal kinase (JNK), anti-JNK, anti-CDK2, anti-CDK4, anti-cyclin D1, anti-cyclin E1, anti-phospho-retinoblastoma protein (Rb), anti-proliferating cell nuclear antigen (PCNA) (PC10), and anti-β-actin antibodies were purchased from Cell Signaling Technology Inc. (Beverly, MA, USA). Cell Counting Kit-8 (CCK-8) was purchased from Dojindo Molecular Technologies (Rockville, MD, USA). Platelet-derived growth factor (PDGF)-BB was obtained from KOMABIOTECH (Seoul, Korea). All other chemicals were of analytical grade.

### Animals

Male LDL-receptor knockout (Ldlr KO, strain name: LdB6.129S7-*Ldlr*^*tm1Her*^/J, C57BL/6 background) mice were obtained from The Jackson Laboratory (Bar Harbor, ME, USA) and acclimated for 1 week at a temperature of 25 ± 2 °C, humidity of 55 ± 5 % and 12:12 h light–dark cycle. After the mice in the negative control group were given unlimited access to AIN-76A (AIN-76A: casein 200, DL-methionine 3, cornstarch 150, sucrose 500, fiber 50, corn oil 50, AIN mineral mix 35, AIN vitamin mix 10, choline bitartrate 2 g/kg) for 2 weeks, they were then fed a Western diet for 14 weeks (fat 21 %, cholesterol 0.15 %, no cholate). The mice in the Kiom-18 group (1 g/kg) were fed a Western diet by gavage (oral administration) and were fed at the same time daily. The animal experiments were conducted in accordance with the Korea Institute of Oriental Medicine Care Committee Guidelines and were accepted by the Korea Institute of Oriental Medicine (KIOM) Care and Use Committee. The animals were cared for in accordance with the dictates of the National Animal Welfare Law of Korea.

### Preparation of the Kiom-18 extract

A total of 2470 g Kiom-18, including *C. cassia, P. densiflora, C. longa* and *G. glabra* (Individual ratio = 1.3 : 1.3 : 1.4 : 1)*,* was placed in 25 L of distilled water and then extracted by heating for 3 h at 115 °C (Gyeongseo Extractor Cosmos-600, Inchon, Korea). The extract of Kiom-18 was filtered using a standard test sieve of 150 μm (Retsch, Haan, Germany), freeze-dried and maintained by desiccators at 4 °C until use.

### Cell culture

Rat aortic VSMCs, isolated by enzymatic dispersion, were obtained from Biobud Co. (Seongnam-si, Gyeonggi-do, Korea). The isolation of VSMCs has been previously reported [46]. The cells were cultured in DMEM (supplemented with 10 % FBS, 100 IU/mL penicillin, 100 μg/mL streptomycin, 8 mM HEPES, and 2 mM L-glutamine) at 37 °C in a humidified atmosphere of 95 % air and 5 % CO_2_. The purity of the cultures was confirmed based on the immunocytochemical localization of α-smooth muscle actin. Our experiment used the VSMCs of passages 5–7.

### Cell proliferation assay

Cell proliferation was measured as previously described [47]. VSMCs were examined using colorimetric WST-1 assays (CCK-8; Dojindo Molecular Technologies, Rockville, MD, USA). VSMCs were seeded into 96-well culture plates at 4 × 10^4^ cells/mL and then cultured in complete media (DMEM containing 10 % FBS) at 37 °C for 24 h. After reaching approximately 70 % confluence, VSMCs were incubated with serum-free medium for 24 h, treated with Kiom-18 of various concentrations for another 24 h in new fresh serum-free medium, and stimulated by PDGF-BB at a concentration of 25 ng/mL. After 24 h, WST-1 reagent (WST-1 premix) was added to the medium, and the cells were incubated for an additional 2 h. The absorbance was measured at 450 nm using a microplate reader (Packard Instrument Co., Downers Glove, IL, USA). All experimental procedures were performed as per the manufacturer’s instructions, and the results are expressed as the percentage of the PDGF-BB-stimulated control.

### DNA synthesis

The measurement of DNA synthesis was monitored using a [^3^H]-thymidine incorporation assay, as previously described [48]. The conditions of this assay were as described for the cell proliferation assay. [^3^H]-thymidine (2 μCi/ml) was added 4 h before harvesting under conditions stimulated by PDGF-BB 25 ng/ml in serum-free medium. The reaction was stopped by aspirating the medium and subjecting the cultures to sequential washes with PBS containing ethanol/ether (1:1, v/v) and 10 % trichloroacetic acid on ice. Acid-insoluble product with [^3^H]-thymidine was extracted in 250 μl of 0.5 M NaOH/well, and this solution was combined with 3 ml of scintillation cocktail (Ultimagold, Packard Bioscience, Meriden, CT, USA) and measured using a liquid scintillation counter (LS3801, Beckman, Düsseldorf, Germany).

### Cell cycle progression analysis

The cell cycle progression was measured as previously described [49]. This assay procedure was the same as that described for the cell proliferation assay. After stimulation with PDGF-BB 25 ng/mL for 24 h, the harvested cells were suspended in 1 mL of PBS and fixed with 70 % ethanol for 48 h. In the fixed cells, the ethanol was removed, and the pellets were then stained with propidium iodide (PI) solution (50 μg/mL PI in sample buffer containing 100 μg/mL RNase A). Each sample was incubated at room temperature for 1 h. The PI-DNA complex of each cell nucleus was measured using a FACSCalibur flow cytometer (Becton, Dickinson and Co., Franklin Lakes, NJ, USA). The individual nuclear DNA content was determined by the incorporated PI fluorescence intensity. The rate of the cell cycle at the G_0_/G_1_, S, and G_2_/M phases was determined by analysis using Modfit LT software (BD, Topsham, ME, USA).

### Immunoblotting

Protein measurement by immunoblotting was performed as previously described [50]. VSMCs were stimulated with PDGF-BB (25 ng/mL) for 5 min for ERK1/2 and PLCγ1, 10 min for JNK and p38, and 15 min for Akt phosphorylation assays. For the measurements of CDK2, CDK4, cyclin D1, cyclin E_1_, p27 and PCNA expression, and Rb phosphorylation, VSMCs were stimulated with PDGF-BB (25 ng/mL) for 24 h. Each of the proteins were normalized with β-actin or the respective total proteins. The band intensities were quantified using Scion Image for Windows (Scion Corp., Frederick, MD, USA).

### Immunofluorescence staining

VSMCs were cultured in 24-well plates that included cover slips at 8 × 10^4^ cells/mL and were then stimulated with PDGF-BB 25 ng/mL for 24 h, after which they were fixed with 2 % paraformaldehyde in PBS for 10 min and then blocked with 5 % bovine serum albumin in PBS for 1 h at room temperature. The cells were stained with primary antibodies (anti-p27) for 2 h and then with Alexa Fluor 555-conjugated secondary antibody (Cell Signaling Technology Inc., Beverly, MA, USA). The cells were mounted in PI solution for 10 min and observed using a fluorescence microscope (TH4-200; Olympus Optical Co. Ltd., Tokyo, Japan).

### Atherosclerotic lesion analysis

The mice were anesthetized with an intraperitoneal injection of ketamine (50 mg/kg) and xylazine (6.7 mg/kg). After perfusion-fixing with 10 % buffered formalin, the aortic arches were transversely sectioned and stained with hematoxylin and eosin (H&E) as previously described [51, 52].

### Plasma lipid analysis

A Hitachi 7080 (Tokyo, Japan) biochemical analyzer was used to measure the serum concentrations of triglyceride (TG), aspartate aminotransferase (AST), alanine aminotransferase (ALT), glucose, total cholesterol (T-C), high-density lipoprotein-cholesterol (HDL-C) and low-density lipoprotein-cholesterol (LDL-C) as previously described [53, 54].

### Statistical analysis

The data are expressed as the mean ± SEM. One-way ANOVA was used for multiple comparisons (GraphPad, San Diego, CA, USA). Dunnett’s test was applied if a significant difference was observed among the treated groups. A value of *p* < 0.05 was considered statistically significant.

## Results

### Effect of Kiom-18 on VSMC proliferation and early signal phosphorylation

**Fig. 1:**
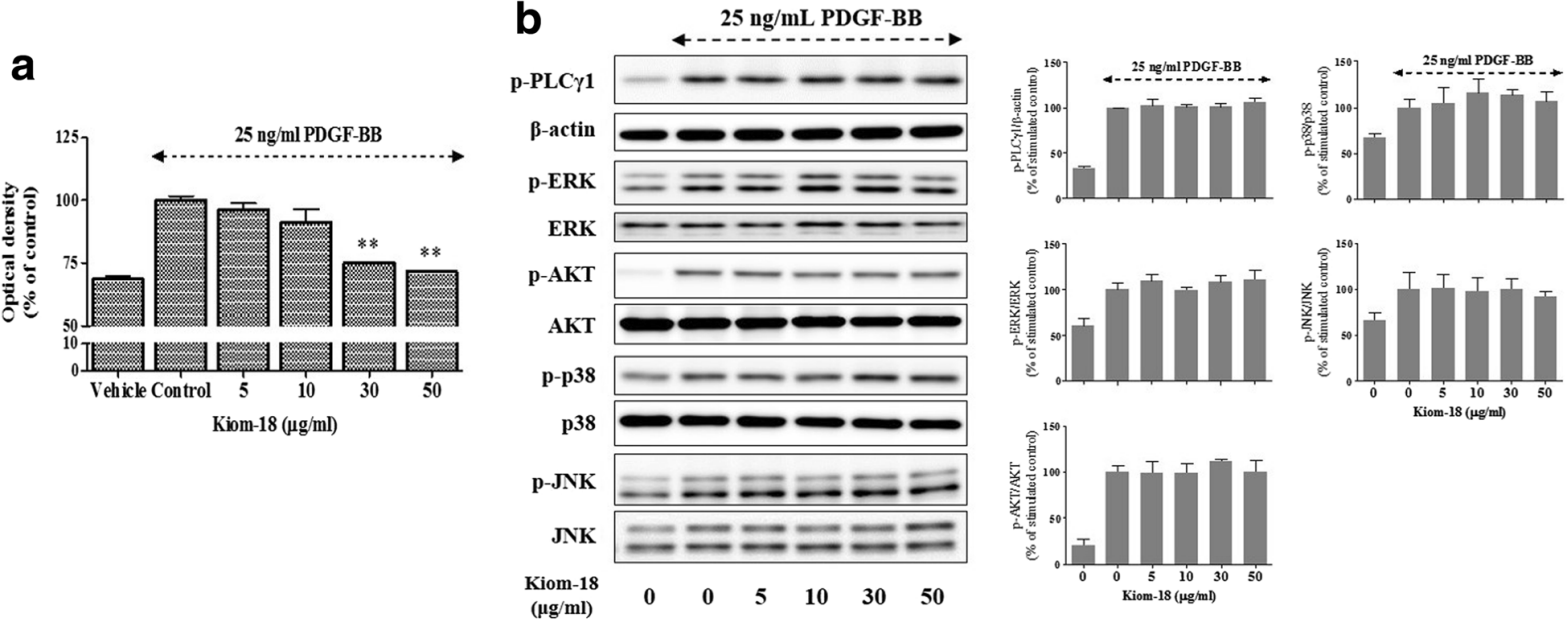
Effect of Kiom-18 extract on VSMC proliferation and early signal transduction. VSMCs cultured in serum-free medium were stimulated with 25 ng/mL PDGF-BB for 24 h, and the effect of various concentrations of Kiom-18 extract (5–50 μg/ml) was monitored. **a** Optimal densities were determined at 450 nm using the WST-1 assay (*n* = 4). **b** The PDGF-induced phosphorylation of PLCγ1, ERK1/2, Akt, p38, and JNK was measured using SDS-PAGE and immunoblotting. β-actin, ERK1/2, Akt, p38, and JNK were used for normalization (*n* = 3). These results were analyzed using colorimetry (**a**) and densitometry (**b**); the values represent the percentage of the control stimulated by PDGF-BB. The values represent the mean ± SEM. Significant differences from the PDGF control (PDGF-stimulated) are shown by **p < 0.01

To determine the effect of Kiom-18 extract on VSMC proliferation, a colorimetric WST-1 assay was performed. Kiom-18 extract at a concentration of 30 and 50 μg/ml significantly inhibited VSMC proliferation compared with the PDGF-BB-stimulated control (Fig. 1a). To identify the effect of Kiom-18 on VSMC proliferation via early signal transduction pathways, we examined the activation of PLCγ1, Akt, p38, ERK1/2, or JNK; however, no difference was observed in the phosphorylation of early signal pathways in the presence of Kiom-18 (Fig. 1b). These results suggest that Kiom-18 has anti-proliferative activity through its downstream effects, although not with regard to early signal transduction pathways, such as the arrest of cell cycle progression, the suppression of cell cycle-related proteins, or the expression of cyclin-dependent kinase inhibitors (CKIs).

### Effect of Kiom-18 on DNA synthesis and cell cycle progression

**Fig. 2:**
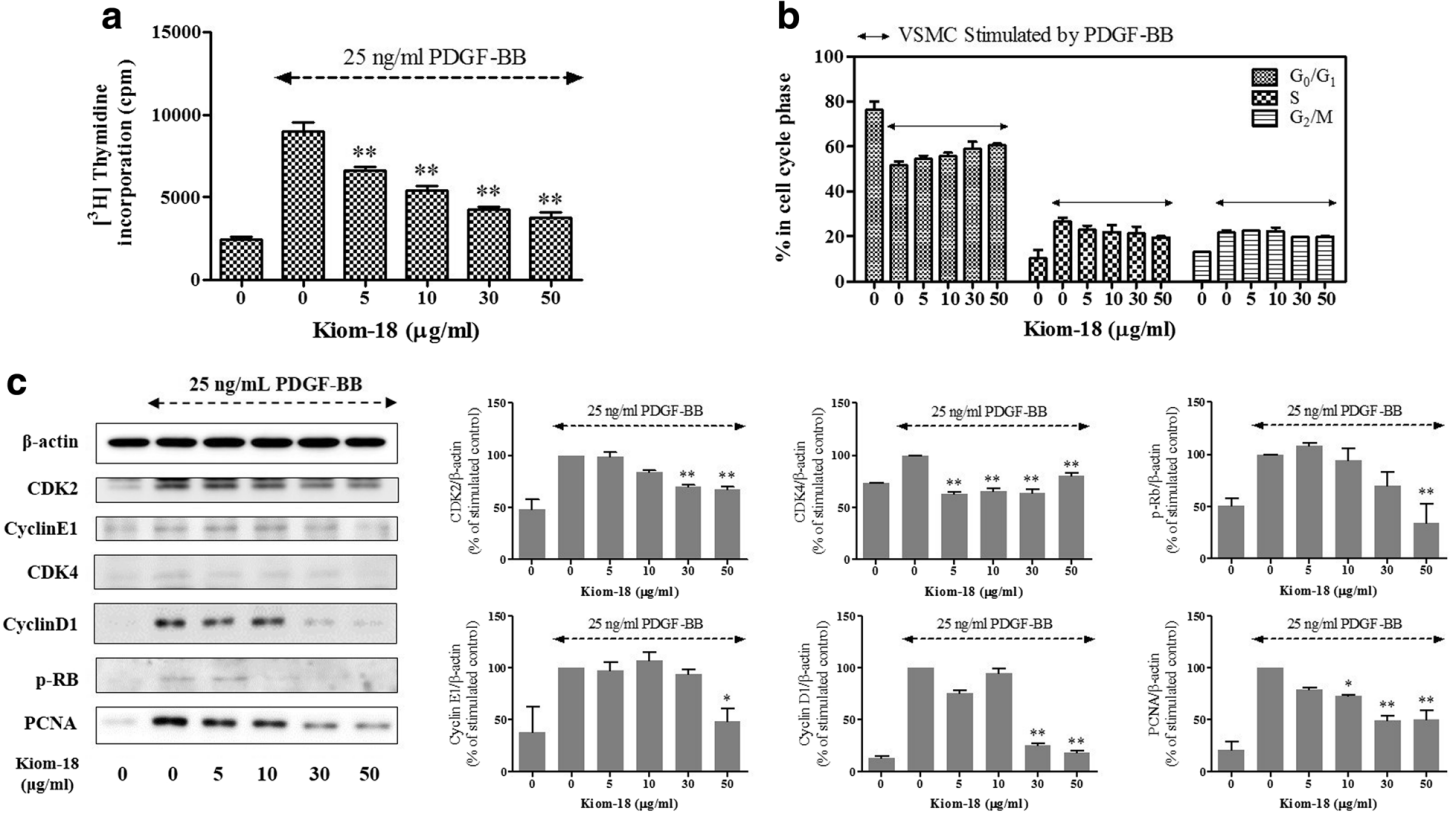
Effect of cinnamon extract on cell cycle progression and cell cycle-related proteins. VSMCs cultured in serum-starved medium were stimulated with 25 ng/mL PDGF-BB and the effect in the presence of Kiom-18 extract (5–50 μg/ml) on the DNA synthesis (**a**) and the histogram data (**b**) for each phase of the cell cycle is shown. The cell cycle progression data are representative of three independent experiments. Moreover, the effect of Kiom-18 extract on cell cycle regulatory proteins stimulated by PDGF-BB, including cyclin D1/E1, CDK2/4, Rb, and PCNA as negative regulatory molecules (**c**), was measured using SDS-PAGE followed by immunoblotting. Total β-actin was used for normalization. These results were analyzed using densitometry; the values represent the percentage of the control stimulated by PDGF-BB. All values are expressed as the mean ± SEM (*n* = 3). Significant differences from the PDGF control (PDGF-stimulated) are shown by *p < 0.05 or **p < 0.01

By [^3^H]-thymidine incorporation, Kiom-18 extract at concentrations of 5, 10, 30, and 50 μg/ml suppressed by 26.7 ± 2.5, 40.0 ± 3.4, 52.9 ± 2.2, and 58.6 ± 3.7 % compared to the PDGF-BB-stimulated control, respectively (Fig. 2a). The results shown in Fig. 2b demonstrate that cell cycle progression was synchronized by the addition of PDGF-BB. This result indicates the arrest of the cell cycle at the transition from G_0_/G_1_ to the S phase after treatment with Kiom-18 extract because the result matched the inhibition effect on DNA synthesis.

### Effects of Kiom-18 on cell cycle-related proteins and p27 expression

**Fig. 3:**
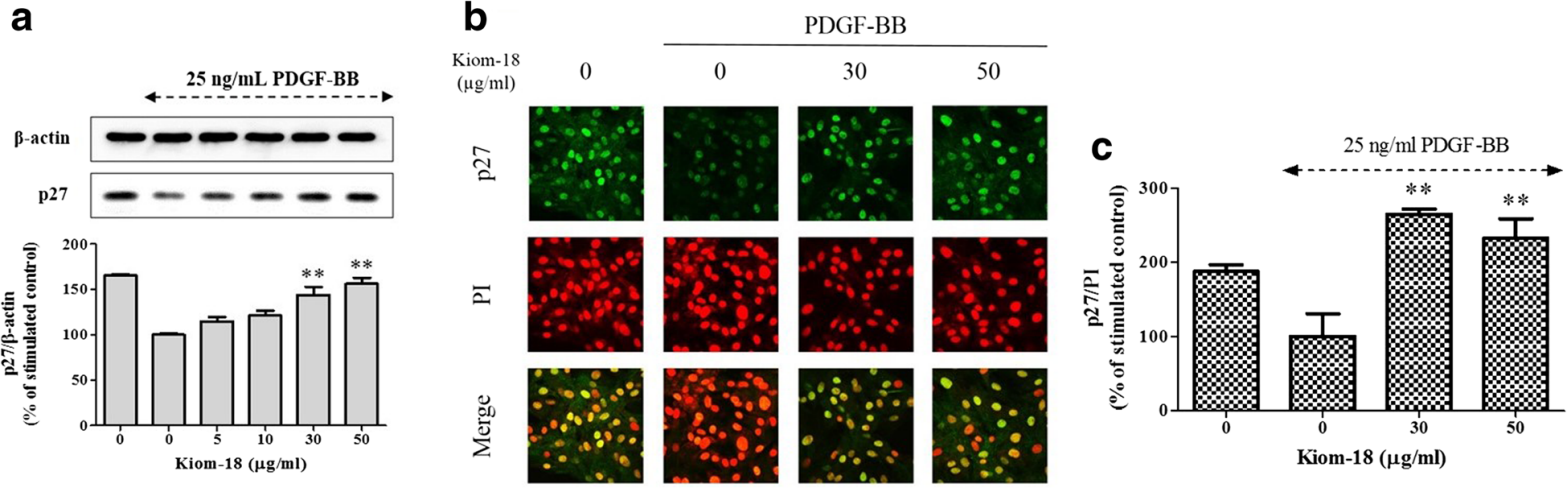
Effect of Kiom-18 extract on the degradation of p27^Kip1^ expression stimulated by PDGF-BB. Quiescent VSMCs cultured in serum-deprived medium were stimulated with 25 ng/mL PDGF-BB, and the inhibitory effect in the presence of Kiom-18 extract (5–50 μg/ml) on p27^Kip1^ expression was examined using SDS-PAGE followed by immunoblotting (**a**), and immunofluorescence (**b**). Total β-actin and the fluorescence level of PI were used for normalization. The immunoblotting (**a**) and immunofluorescence (**c**) were analyzed using densitometry; the values represent the percentage of the stimulated control or the non-stimulated control. The results are an average of three similar, independent experiments and are expressed as the mean ± SEM. Significant differences compared to the control stimulated by PDGF-BB are shown by **p < 0.01

To determine the molecular mechanism with regard to the inhibitory effect of Kiom-18 on cell cycle progression, we investigated the inhibitory effect of Kiom-18 extract on CDK2, CDK4, cyclin D1, cyclin E1, PCNA expression and Rb phosphorylation as positive regulatory proteins. As shown in Fig. 2c, Kiom-18 significantly inhibited the expression of CDK 2/4 and cyclin D1/E1, the G_0_/G_1_ phase-related proteins. Kiom-18 also suppressed Rb phosphorylation, which is a major component of the molecular network that modulates the restriction point in the cell cycle progression. Subsequently, the presence of the Kiom-18 extract inhibited PCNA, which is synthesized by a gene that is triggered by hyper-phosphorylated Rb in the early G_0_/G_1_ and the S phases (Fig. 2c). We next investigated the effect of Kiom-18 extract on p27 expression. Figure 3a shows that Kiom-18 concentration-dependently increased the expression of p27, which is the negative regulatory protein of kinase and cyclin, thereby arresting the cell cycle at the G_0_/G_1_ phase [55].

Additionally, based on the immunofluorescence level of p27 expression, Kiom-18 extract at doses of 30 and 50 μg/ml increased by approximately 2.6- and 2.3-fold, respectively, compared to the PDGF-BB-stimulated control (Fig. 3b and c). This result, along with the immunoblot results, demonstrates that the cell cycle at the G_0_/G_1_ phase was suppressed by the presence of the Kiom-18 extract.

### Effects of Kiom-18 on LDL-receptor knockout mice

**Fig. 4:**
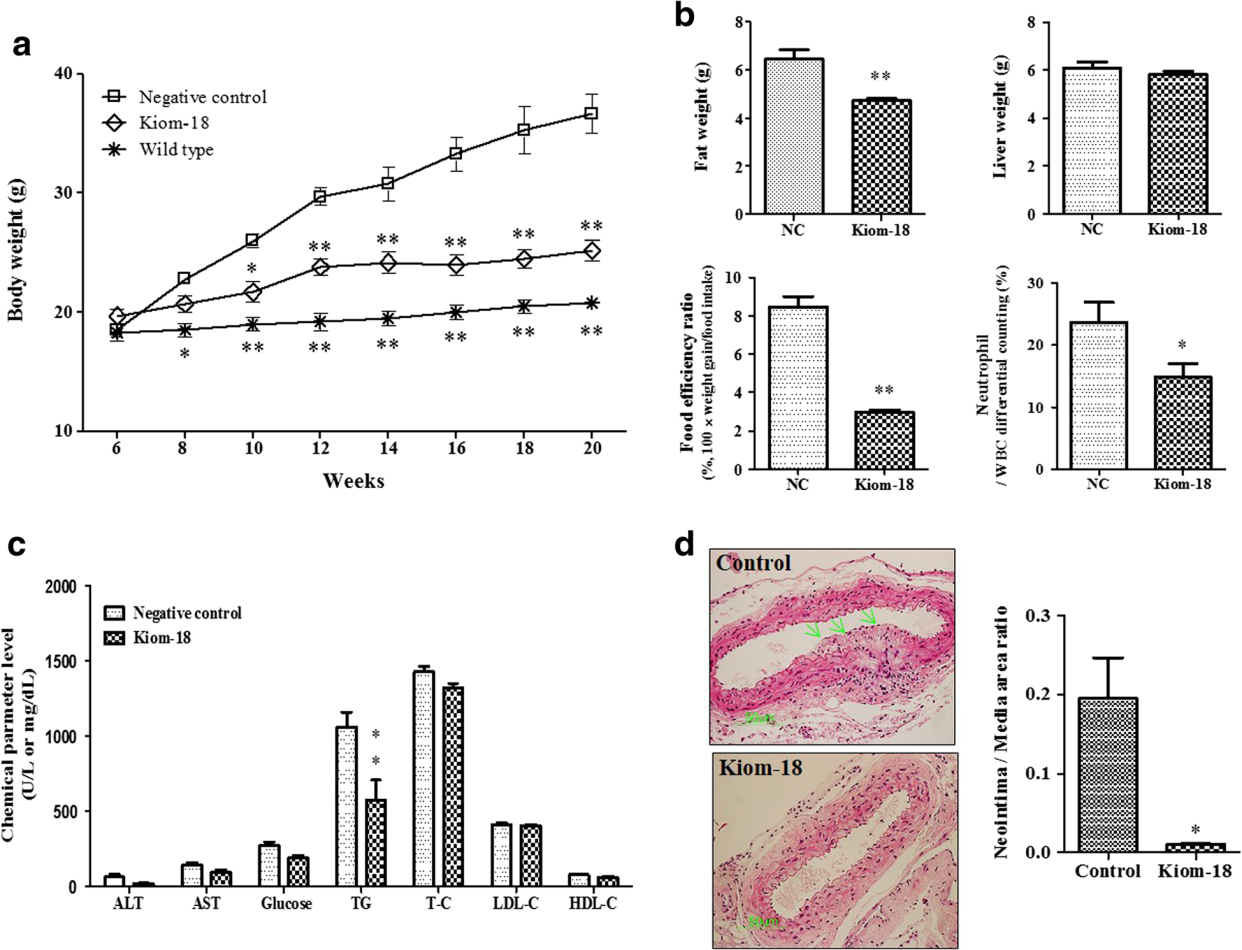
Effect of Kiom-18 extract on the formation of atherosclerotic plaque. The negative control (NC) mice were given unlimited access to a normal diet (AIN-76A) for 2 weeks, and then they were fed a Western diet for 14 weeks (*n* = 4). The treatment group of mice were administered Kiom-18 daily by gavage at a concentration of 1 g/kg and were fed a Western diet. The mice were measured for body weight (**a**), fat weight, liver weight, food efficiency, neutrophils (**b**), and plasma biochemical parameters (**c**). Atherosclerotic plaque and neo-intima/media area ratio were analyzed in the transverse section stained with H&E (**d**). The data are presented as the mean ± SEM. Significant differences compared to the negative control (NC) are shown by *p < 0.05 or **p < 0.01

We used LDLr knockout mice to identify the immediate effect of Kiom-18 extract in an atherosclerosis animal model. The body weight of the mice fed a Western diet for 14 weeks increased; however, that of the group administered Kiom-18 extract significantly decreased (Fig. 4a). As shown in Fig. 4b, Kiom-18 extract also suppressed the fat weight (g), the food efficiency ratio (100 × weight (g)/food intake (g)) [56], and neutrophils (%). Additionally, the serum triglyceride (TG) level was significantly inhibited by Kiom-18 extract compared with the negative controls (Fig. 4c). These results suggest that Kiom-18 extract has a suppressive effect on the atherosclerotic conditions induced by Western dietary consumption, including hyperlipidemia, inflammation, and obesity. In the analysis of the atherosclerotic lesions, Kiom-18 showed an inhibitory effect on neo-intimal formation via VSMC hyperplasia in the aortic arch (Fig. 4d). Moreover, Kiom-18 significantly reduced the neo-intima/media area ratio compared with the control. These findings indicate that the inhibition effect of Kiom-18 is due to the anti-proliferative activity of VSMCs as a result of the events described above.

## Discussion

A large number of functional food products, several of which have shown efficacy, have been developed and marketed [57, 58]. To develop a functional supplement (food) to prevent cardiovascular diseases, such as atherosclerosis, restenosis, stroke and myocardial infarction, we conducted screening tests of traditional medicine stored at the Korea Institute of Oriental Medicine. As a result, Kiom-18, which is a novel composition, was produced as a functional supplement with considerations for safety, efficacy and the supply of raw materials. The Kiom-18 patent has been registered to gain the recognition of the Korean Intellectual Property Office (KIPO) regarding the novelty and efficacy of this composition (registration number: KR1461588).

In this study, we demonstrated that Kiom-18, a novel composition of *C. cassia, P. densiflora, C. longa* and *G. glabra*, prevents atherosclerosis through the anti-proliferative effect on VSMCs via the regulation of cell cycle-related proteins such as CDK2, CDK4, cyclin D1, cyclin E1, Rb, and PCNA. These effects of the Kiom-18 extract were identified as preventive by increasing p27 expression, which has been recognized as a negative regulator of cyclins and protein kinase, and in arresting the cell cycle at the G_0_/G_1_ phase [13, 55]. Although it will require further study, including in clinical trials, Kiom-18 extract may be used as a functional food supplement to manage atherosclerosis.

Atherosclerotic lesion formation is caused by VSMC proliferation stimulated by cytokines and growth factors secreted via endothelial cell dysfunction including the inflammatory process [59, 60]. Ultimately, this pathway is modulated by the transcriptional process of cell cycle-regulatory proteins and genes promoting the proliferation of VSMCs [61]. Hence, the inhibition of VSMC proliferation is considered an important target for the treatment and prevention of atherosclerosis.

In the present study, we confirmed the anti-proliferative activity of Kiom-18 extract on VSMCs, which significantly inhibited PDGF-BB-stimulated VSMC proliferation (Fig. 1a). VSMC proliferation was primarily regulated by the phosphorylation of early signals, such as AKT, ERK1/2, JNK, p38, and PLCγ1, stimulated by PDGF-BB [49, 62]. However, in this study, Kiom-18 extract had no direct effect on the phosphorylation of early signals, suggesting that the anti-proliferative activity of Kiom-18 extract was not due to its effect on early signaling pathways (Fig. 1b). We next examined the effect of Kiom-18 in the downstream signal transduction of early signal phosphorylation, including cell cycle progression, to determine the target of its anti-proliferative effect [63]. Cell cycle progression in VSMC proliferation is modulated by regulatory proteins, such as cyclins, CDKs, pRb, and PCNA, which are considered important targets for the inhibition of proliferation [11, 61, 64, 65]. Kiom-18 extract concentration-dependently suppressed DNA synthesis via cell cycle arrest at the G_0_/G_1_ phase (Fig. 2a and b). Among the sequential phases of the cell cycle, the transition from the G_0_/G_1_ phase to the S phase was mediated by CDK2/cyclin E1 and CDK4/cyclin D1 complexes [66]; as a result, this transition led to the phosphorylation of Rb. The Kiom-18 extract, according to the findings regarding cell cycle progression, significantly suppressed the phosphorylation of Rb by inhibiting the CDK2/cyclin E1 and CDK4/cyclin D1 complexes, resulting in the decreased expression of PCNA (Fig. 2c), which is synthesized as a product of the phosphorylated Rb-mediated gene in the cell cycle transition of G_0_/G_1_ to S phase [67]. This transition at G_0_/G_1_ to S phase was modulated by the CKIs such as INK4 and the Cip/Kip family [11]. Among the Cip/Kip family, the Kiom-18 extract increased the expression of p27^Kip1^ in the immunoblotting assay (Fig. 3a), as it is a negative regulator associated with protein kinase and cyclin of the cell cycle at the G_0_/G_1_ phase [55]. Additionally, p27^Kip1^ expression was identified by an increase in the fluorescence level while in the presence of the Kiom-18 extract (Fig. 3b), which was significantly upregulated in correlation with the immunoblotting results (Fig. 3c). These results indicate that the anti-proliferative effect of Kiom-18 extract is associated with the arrest of the cell cycle transition of G_0_/G_1_ to S phase via the upregulation of p27^Kip1^ expression. This anti-proliferative activity of Kiom-18 extract is caused by the modulation of cell cycle-related proteins rather than by early signal transduction pathways [48, 68].

The enhanced proliferation of VSMCs contributed to the formation of atherosclerotic plaque [69], and although the KIOM-18 extract was used in a relatively high concentration in LDLr knockout mice, the Kiom-18 extract suppressed the development of atherosclerotic plaque in an animal model (Fig. 4). In previous studies using p27^Kip1^ or ApoE-deficient mice, p27^Kip1^ expression was reported to be an important factor in preventing atherosclerosis [70, 71]. Even if p27^Kip1^ expression was not directly identified in animal models, based on previous reports, the inhibitory effect of Kiom-18 on the formation of atherosclerotic plaque could be expected by the upregulation of p27^Kip1^ expression. Moreover, because the development of atherosclerotic plaque is caused by the consumption of a high-fat diet, this animal model demonstrated increases in body weight, fat weight, liver weight, food efficiency, neutrophil count, and blood chemical parameters. The Kiom-18 extract significantly inhibited the body weight (Fig. 4a), fat weight, food efficiency ratio (%), neutrophils (by % of WBC differential counting) (Fig. 4b), and TG level (Fig. 4c). These results indicate that Kiom-18 extract has preventive effects in a high-fat-induced atherosclerosis animal model.

## Conclusion

Our results demonstrate that Kiom-18 extract inhibited the formation of atherosclerotic plaque by inhibiting VSMC proliferation through a G_0_/G_1_ arrest, which upregulated the p27^Kip1^ expression. Therefore, we cautiously anticipate that Kiom-18 extract may be used as a functional food supplement or as a preventive agent to manage atherosclerosis.

## Abbreviations

Akt, phosphatidylinositol 3-kinase-linked protein kinase; *C. cassia*, *Cinnamomum cassia*; *C. longa*, *Curcuma longa*; CCK-8, cell counting Kit-8; CDK, cyclin-dependent kinase; CKI, cyclin-dependent kinase inhibitor; DMEM, Dulbecco’s modified Eagle’s medium; ERK, extracellular signal regulated kinase 1/2; FBS, fetal bovine serum; *G. glabra*, *Glycyrrhiza glabra*; JNK, c-Jun N-terminal kinase; LDL, low-density lipoprotein; *P. densiflora*, *Pinus densiflora*; PBS, phosphate-buffered saline; PCNA, proliferating cell nuclear antigen; PDGF-BB, platelet-derived growth factor; PLCγ1, phospholipase C-γ1; Rb, retinoblastoma protein; VSMC, vascular smooth muscle cell
